# Abdominal Wall Haematoma Complicating Laparoscopic Cholecystectomy

**DOI:** 10.1155/1994/31586

**Published:** 1994

**Authors:** S. Bhattacharya, J. J. T. Tate, B. R. Davidson, K. E. F. Hobbs

**Affiliations:** University Department of Surgery Royal Free Hospital and School of Medicine Pond Street London NW3 2QG UK

## Abstract

Of 61 consecutive patients undergoing laparoscopic cholecystectomy, 4 (6.25%) developed abdominal wall haematomas. This complication of laparoscopic cholecystectomy may occur more commonly than existing literature suggests, and manifests in the post-operative period (days 2 to 6) by visible bruising, excessive pain or an asymptomatic drop in haematocrit. It is readily confirmed by ultrasonography. While no specific treatment is necessary apart from replacement of significant blood loss, the patient requires reassurance that this apparently alarming complication will rapidly resolve.


*HPB Surgery*, 1994, Vol. 7, pp. 291–296
Reprints available directly from the publisher
Photocopying permitted by license only

© 1994 Harwood Academic Publishers GmbH
Printed in the United States of America

### ABDOMINAL WALL HAEMATOMA COMPLICATING LAPAROSCOPIC CHOLECYSTECTOMY

S. BHATTACHARYA, J. J. T. TATE, B. R. DAVIDSON and K. E. F. HOBBS

*University Department of Surgery, Royal Free Hospital and School of Medicine, Pond Street, London NW3 2QG, U.K.*

Of 61 consecutive patients undergoing laparoscopic cholecystectomy, 4 (6.25%) developed abdominal wall haematomas. This complication of laparoscopic cholecystectomy may occur more commonly than existing literature suggests, and manifests in the post-operative period (days 2 to 6) by visible bruising, excessive pain or an asymptomatic drop in haematocrit. It is readily confirmed by ultrasonography. While no specific treatment is necessary apart from replacement of significant blood loss, the patient requires reassurance that this apparently alarming complication will rapidly resolve.

KEY WORDS: Laparoscopic Cholecystectomy, Complication, Haematoma, Abdominal Wall.

### INTRODUCTION

Laparoscopic cholecystectomy is now accepted in most centres as the treatment of choice for symptomatic gallbladder stones. However, there is an increasing awareness among surgeons of the associated complications. Diagnostic laparoscopy itself is associated with morbidity rates of 0.15% to 0.6%, many of the complications arising from creation of the initial pneumoperitoneum or insertion of the trocar[1]. Review of the haemorrhage-related complications in several large series of laparoscopic cholecystectomies[2–6] suggests that while intra-operative bleeding from the abdominal wall vessels is well recognised, late presentation with abdominal wall haematoma is rare. We report our experience with four cases of laparoscopic cholecystectomy complicated by abdominal wall haematomas.

### METHODS AND RESULTS

Over a period of one year, 61 laparoscopic cholecystectomies were performed by 3 operating surgeons familiar with laparoscopic technique. Standard equipment was used in all cases and the ports were sited in the usual fashion (one 10 mm subumbilical transverse, one 10 mm epigastric midline, and two 5 mm ports in the right upper

---

Correspondence to: Mr. S. Bhattacharya quadrant). Electro-diathermy was used for haemostasis. Four patients developed abdominal wall haematomas in the post-operative period, and their presentation and management is outlined below (see Table).

*Patient No. 1*

A 30 years old woman with acute biliary colic underwent laparoscopic cholecystectomy after ultrasonography revealed multiple small stones in a thick-walled gallbladder. On the 2nd post-operative day, she complained of pain in the right flank radiating to the shoulder which subsided with oral analgesia. She was discharged on day 4, but returned to the hospital casualty department on day 6, with further pain in the right flank and iliac fossa. Ultrasonography showed an abdominal wall haematoma between the right external and internal oblique muscles. She was treated with analgesics as an out-patient, and recovered without further complications.

*Patient No. 2*

This young woman of 29 with mild acute pancreatitis was shown on ultrasonography and ERCP to have multiple gallbladder stones and a normal common bile duct. Laparoscopic cholecystectomy performed 7 days after admission was uneventful. Her haemogobin on the 2nd post-operative day showed a drop of 3 g/dl. Ultrasonography revealed two large haematomas in the anterior abdominal wall, one periumbilical and the other suprapubic in location. No free fluid was noted in the peritoneal cavity. She was transfused with 3 units of blood and discharged on the 4th day after surgery.

*Patient No. 3*

An obese woman aged 57 presented with a long history of right hypochondrial discomfort. Ultrasonography revealed a thick-walled gallbladder with numerous stones and elective laparoscopic cholecystectomy was performed. The procedure lasted over 2 hours and fibrotic scarring in the gallbladder bed rendered dissection difficult, with more than average bleeding. Satisfactory haemostasis was achieved, and a suction drain was left in the subhepatic space for 24 hours. The patient was discharged on the 2nd post-operative day, but returned on day 5, with persistent upper abdominal discomfort, remarkable bruising of the abdominal wall (see figure), and mild jaundice. Her haemoglobin had fallen by 4 g/dl, and her liver function tests (LFTs) showed a raised bilirubin with normal transaminases and alkaline phosphatase, compatible with haemolysis. Abdominal ultrasound showed a fluid collection (haematoma) in the right subphrenic space. She was readmitted and transfused with 4 units of blood. No other active measures were required; her symptoms gradually subsided, with resolution of the haematoma over 5 days.

*Patient No. 4*

A fit man of 68 investigated for chronic epigastric pain was found to have a solitary 4 cm stone impacted in the neck of a thick-walled gallbladder on ultrasonography. The

**Table** Summary of presentation and management of the four patients

| *Age/Sex & physical stature* | *Indication for surgery* | *Findings on Ultrasound before surgery* | *Operative findings* | *Haematoma detected* | *Principal symptom* | *Visible bruise on abdominal wall* | *Haematoma as seen on Ultrasound* | *Drop in Haemoglobin* | *Changes in Liver function tests* |
|---|---|---|---|---|---|---|---|---|---|
| 30/Female (normal build) | Acute biliary colic | Thick-walled gallbladder with multiple stones | Inflamed gallbladder; Omental adhesions | Postoperative day 6 (after discharge from hospital) | Pain | No | Intramural | Nil | None |
| 29/Female (normal build) | Biliary pancreatitis | Gallbladder stones (Bile duct normal on ERCP) | Thin-walled gallbladder | Postoperative day 2 (prior to discharge from hospital) | No symptoms | No | Intramural | 3 g/dl (given blood transfusion) | None |
| 57/Female (obese) | Chronic cholecystitis | Thick-walled gallbladder with multiple stones | Fibrotic gallbladder; difficult dissection; | Postoperative day 5 (after discharge); Required re-admission | Pain Mild jaundice Bruising | Remarkable bruising | Intramu-ral + sub--phrenic | 4 g/dl (given blood transfusion) | Raised Bilirubin; Normal ALP, AST |
| 68/Male (normal build) | Chronic cholecystitis | Thick-walled gallbladder with solitary 4 cm stone | Fibrotic gallbladder; difficult dissection; | Postoperative day 5 (after discharge from hospital) | Pain | Prominent bruises | Subphrenic | Nil | None |

Key: ERCP = Endoscopic Retrograde Cholangio-Pancreatography ALP = Alkaline Phosphatase AST = Aspartate Aminotransferase.

elective laparoscopic cholecystectomy lasted 2 hours and the dissection in the gallbladder area was more difficult than usual. He was discharged on the 2nd post-operative day, but returned on day 5, with significant epigastric pain radiating to the right shoulder and marked bruising of the anterior abdominal wall. His haematocrit and LFTs were normal, and ultrasound showed a small haematoma in the right subphrenic space. He was reassured, and the symptoms abated over the next few days without requiring hospitalisation.

### DISCUSSION

Our experience would suggest that abdominal wall haematoma is a common complication of laparoscopic cholecystectomy, occurring in 6.25% of patients in this series. It presents between the 2nd and 6th days after surgery, and can lead to delay in discharge from hospital, or re-admission following discharge. When manifesting with obvious bruising, it causes considerable anxiety to the patient. Apart from visible ecchymoses, this complication should be suspected in any patient suffering from an unusually high level of post-operative pain, and in asymptomatic patients who show a sudden unexplained drop in haematocrit after surgery. It is remarkable that one of our patients had an intramural haematoma sufficiently large to bring her haemoglobin down by 3 g/dl and yet did not have any symptoms. Mild icterus and abnormal liver function tests, as noted in one of our patients, may be a consequence of gradual lysis of the clot. An ultrasound of the abdomen is the primary imaging investigation, but detects blood within the abdominal wall only if it is specifically looked for. No active treatment was required in any of our cases, apart from an explanation and reassurance. A significant drop in haematocrit should be corrected by blood transfusion, and if necessary, oral iron supplementation.

The incidence of abdominal wall haematomas in this series is much higher than existing reports would lead one to expect. The Southern Surgeons Club, in their report of 1518 cases[2], describe 3 cases (0.2%) requiring transfusion following surgery, and 2 patients suffering excessive post-operative pain, but there is no record of abdominal wall haematomas. Another study[3] from the United States reports 1983 cases, including 5 patients with peri-operative bleeding from the gallbladder area, who were treated with transfusion and observation, and one episode of epigastric artery bleeding following trocar insertion, which was sutured, but again no reference is made to abdominal wall haematomas. Wolfe *et al.*[4], reporting 381 cases, describe one episode of post-operative haemorrhage. A collective report of the European experience[5], which includes 1236 patients, mentions 6 instances (0.5%) of "ecchymoses", but does not elaborate on their presentation and management. An earlier report from Dundee[6] of 61 cases includes one patient requiring post-operative transfusion for a drop in haematocrit. The true incidence of this problem may have been under-reported, as all cases of postoperative pain are not subjected to abdominal ultrasonography, and post-operative haemoglobin estimations are not routinely performed.

Two of our cases (patients 1 & 2) had an intramural haematoma with no evidence of intra-abdominal bleeding, which would suggest that it originated from an abdominal

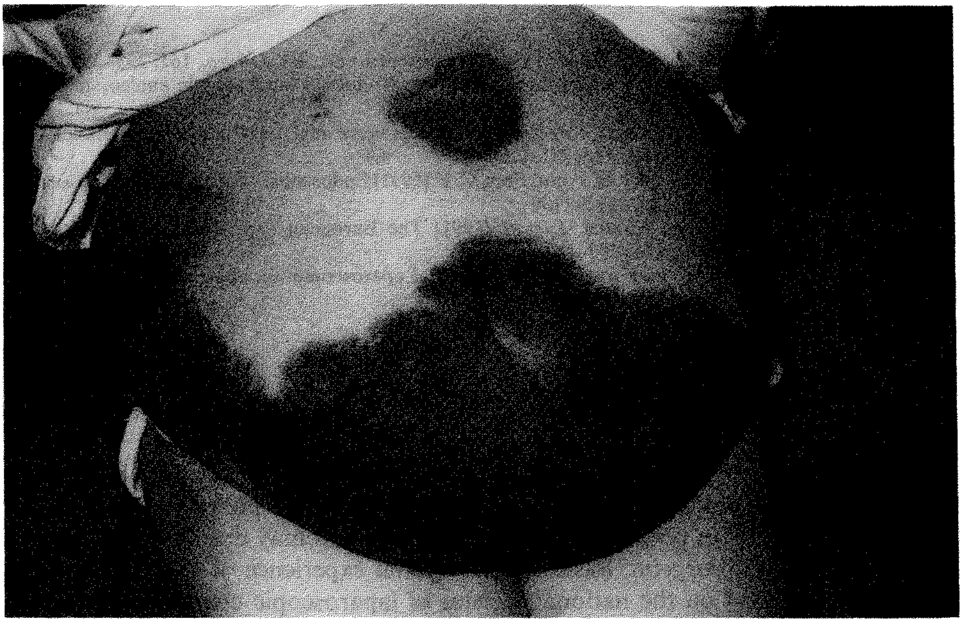

**Figure 1** Abdominal wall haematoma following laparoscopic cholecystectomy.

wall vessel damaged while siting an access port. Haematoma formation from damage to abdominal wall vessels may be reduced by careful siting of the 5 mm access ports in the right upper quadrant. It has been suggested that transillumination of the abdominal wall prior to insertion of trocars may prevent puncture of a medium-sized vessel, and observing the point of entry through the laparoscope for a few minutes after each port has been inserted allows early detection of a bleed. The 5 mm ports were not sutured in our patients, and it may be argued that suturing all port sites would reduce the chances of haematoma formation.

The other two patients had abdominal wall haematomas and visible bruising in conjunction with collections of blood in the right subphrenic space. It is possible that this was caused by reactionary haemorrhage from the gallbladder bed which tracked through multiple access ports into the abdominal wall and also collected under the diaphragm. The distribution of bruising in one of them (see figure), would support this possibility. One of these patients was remarkably obese and both had received subcutaneous heparin as anti-thrombotic prophylaxis. Both had fibrotic, thick-walled gallbladders, requiring prolonged dissection with difficult haemostasis. Dissection of a inflamed gallbladder may predispose to this complication. In situations where significant bleeding has been encountered from the gallbladder area, the placement of suction drains may seem appropriate; but it proved to be of no value in our case (patient 3), and will not compensate for imperfect haemostasis during surgery.

### *References*

1. Ponsky, J. L. (1991) Complications of laparoscopic cholecystectomy. *Am. J. Surg.*, **161**, 393–395
2. The Southern Surgeons Club (1991) A prospective analysis of 1518 laparoscopic cholecystectomies. *N. Engl. J. Med.*, **324**, 1073–1078
3. Larson, G. M., Vitale, G. C. and Casey, J, *et al.* (1992) Multipractice analysis of laparoscopic cholecystectomy in 1983 patients. *Am. J. Surg.*, **163**, 221–226
4. Wolfe, B. M., Gardiner, B. N., Leary, B. F. and Frey, C. F. (1991) Endoscopic cholecystectomy: an analysis of complications. *Arch. Surg.*, **126**, 1192–1199
5. Cuschieri, A., Dubois, F. and Mouiel, J, *et al.* (1991) The European experience with laparoscopic cholecystectomy. *Am. J. Surg.*, **161**, 385–387
6. Nathanson, L. K., Shimi, S. and Cuschieri, A. (1991) Laparoscopic cholecystectomy: the Dundee technique. *Br. J. Surg.*, **78**, 155–159

### INVITED COMMENTARY

It is important to draw attention to side effects by laparoscopic procedures and to inform about the risk of postoperative haematoma which may upset the patient, the nursing staff and the surgeon. Based on a personal experience of 250 laparoscopic operations and based on the national register of laparoscopic cholecystectomies in Sweden, major abdominal wall hematoma is not a frequent problem (10 reported cases out of 4000 laparoscopic cholecystectomies). In my opinion there are also measures to avoid this problem.

Injection of local anaesthetics with epinephrine prior to incision. This will contract the vessels at the introduction of troacars. Further it may reduce postoperative inflammatory response and pain in the abdominal wall.

The injection needle may be used as a probe and its tip may be inspected when it is perforating the peritoneum. This is helpful as a direction for the insertion of troacars.

Incise the port in the skin and debride with a clamp to preform a channel for the troacar. If there is a bleeding from the port incision try to coagulate it early.

Joar Svanvik
Professor of Surgery
University Hospital
Linköping

